# Green effects of research and development on industrial waste reduction during the production phase: Evidence from China and policy implications

**DOI:** 10.3389/fpubh.2022.1000393

**Published:** 2022-10-20

**Authors:** Erzi Tang

**Affiliations:** School of Economics, Nanjing Audit University, Nanjing, China

**Keywords:** industrial waste, research and development (R&D) activities, green development, innovative development, public health

## Abstract

Maintaining public health requires a clean environment; however, some industrial wastes can damage the water, atmosphere, and living environment seriously. To promote green development, policy makers in China have developed and implemented strict environmental regulations to limit the pollutant emissions and improve the environmental quality. Industrial producers implement research and development (R&D) activities to gain more profits in competitive markets. A comprehensive understanding of the green effects of R&D on different industrial wastes could provide important policy recommendations, especially regarding the coordination of innovative and green developments. In this study, the author empirically analyzed the influence of R&D input, including the intramural expenditure on R&D and full-time equivalent of R&D personnel, on industrial wastes, including the discharge of chemical oxygen demand (COD) and ammonia nitrogen, emission of sulfur dioxide, nitrogen oxides, and particulate matter, and generation of common industrial solid and hazardous wastes, based on the data from Chinese industrial sectors for 2016–2020. The main findings of empirical analyses were robust and indicated that R&D activities significantly reduced the emissions of all three industrial waste gases and decreased the discharge of COD; however, in the case of China, the partial effects on the discharge of ammonia nitrogen and the industrial solid wastes were not statistically significant. The green effects of R&D on different industrial wastes may vary and generally depend on environmental regulations, with various limitations. The most viable policy recommendations indicate that by expanding and initiating the green effect of R&D on different industrial wastes, innovative and green developments are more likely to be achieved in a coordinated manner. Additionally, this can also support special R&D activities, with the added benefit of actively developing cleaner technology to treat pollutant emissions. Development, while maintaining a clean environment to ensure public health, could be more sustainable if innovative activities reduce the production of industrial wastes. This study analyzes the green effects of R&D on industrial waste and can serve as a viable framework for future studies on sustainable development.

## Highlights

- Innovative and green developments are the main goals of most public policies.
- Industrial wastes damage environment and threaten public health.
- R&D can reduce some industrial wastes and solve some environmental issues.
- Heterogeneous green effects on different industrial wastes have policy implications.
- Expanding the green effects of R&D on industrial wastes can promote sustainability.

## Introduction

As the biggest developing economy in the world, China has been pursuing economic growth and social development for a long time. Top designers in the state emphasize that people's wish for a good life is the main goal of the Chinese government. In the speech at the press conference of the members of the Standing Committee of the Political Bureau of the 18th Chinese Communist Party (CPC) Central Committee, Xi Jinping, the leader of the CPC and the state, stated that “*Our people have an ardent love for life. They want to have better education, more stable jobs, more income, reliable social security, better medical and health care, improved housing conditions and a beautiful environment*.” Generally, in some developed economies, industrialization and the persistent development of industries drive people to live richer life. In many economies, at the initial stage of economic development, industrialization has changed income distribution and social welfare considerably (1–3). Some developed economies mainly rely on high-tech industry, along with information technology and the service industry, known to have a high quality in the contemporary world; however, traditional industrial departments still play an important role in economic development. Compared with western developed economies, the economic growth and per capita income increase in developing economies, including China, are significantly dependent on the development of different industries. People in developed and developing countries seek a prosperous life. Overall, industrialization produced a great deal of material wealth, but it also caused social problems, e.g., massive consumption of energy and the destruction of ecological environments. A close nexus between natural resources and production exists in many sectors (4); therefore, sustainable management of natural resources and some associated issues are crucial (5). Some regions experienced rapid industrialization and air pollution simultaneously (6). Notably, the public goal of economic prosperity and rapid industrialization resulted in a series of problems, such as the discharge of wastewater contaminated by heavy metals (7). Additionally, in the production phase, industrial solid wastes pose several threats to human health and the environment (8). As industries with high energy consumption and large pollutant emissions play an important role in the economy of China, it is difficult to coordinate energy production and consumption, based on the goals of economic development and environmental protection (9). To achieve high economic growth, some developing countries used massive natural resources and have depleted these resources (10). The supply-demand gaps in natural resources surge in some developing economies (11). As polluted air, water, and land decline the quality of life severely, environmental sustainability is fundamental to human survival (12). Wang et al. (13) suggested that producers in high-energy consumption industries should focus on corporate social responsibility, especially in terms of air pollution. Environment affects public health in a variety of ways (14); therefore, to maintain public health needs to mitigate the harmful impact of human behavior on the environment (15). Economic growth causes environmental pollution and negatively impacts public health in many scenarios; however, the economy has more public resources to improve medical conditions along with economic growth, which also positively influences public health (16). To promote sustainable health for the population, the increasing policies focusing on sustainable development could provide some feasible methods (17), because the nexus between sustainable development and public health is quite strong (18). In economic operation and life environment, wastes from industrial production directly impact public health (19). To ensure a healthy environment, as an important part of a better quality of life, public policies must address issues such as pollution, air quality, and deforestation, along with other associated issues that may result from industrial production.

As a main content in the process of green development, industrial wastes reduction is defined the green effect in this work. Some environmental regulations, implemented through particular public policies, reduce industrial waste and change the structure of industrial production in practice (20). However, some environmental control measures and regulations implemented in China could not control and reduce pollution effectively (21). A better quality of life for the people in many economies entails economic growth; this is especially true for developing countries. The role of energy security in poverty reduction in some poorest economies is also important (22). From a historical perspective, ancient Chinese Confucianism emphasized that policy makers should be responsible for the needs of the people. For example, *The Works of Mencius*, an important Chinese classic, mentioned the following: “*Therefore an intelligent ruler will regulate the livelihood of the people, so as to make sure that, for those above them, they shall have sufficient wherewith to serve their parents, and, for those below them, sufficient wherewith to support their wives and children; that in good years they shall always be abundantly satisfied, and that in bad years they shall escape the danger of perishing*.”^1^ In a hypothetical scenario, if policy makers significantly and directly reduce the output in the industry sector, there will be a consequent reduction in energy consumption and pollutant emissions. However, a direct decline in the industrial output is not the ideal way to solve the environmental issues caused by industrial waste, as this may negatively impact the per capita income and a total output of the economy. To maintain the industrial output, while reducing pollutant emissions and industrial waste, innovative development and technological progress can be regarded as feasible methods. Industrial wastes, mostly formed during the production phase, are derived from the input, rather than from the output; this indicates that higher output and lower pollutant emissions can be achieved when the input per unit output decreases with technological progress. For example, many pollutants are generated from fossil energy consumption (23, 24), and especially in China, industrial production consumes a large part of the energy produced (25). In the last 40 years, the Chinese economy immoderately exploited natural resources for serving growth, caused the ecosystem's degradation and impacted people's health (26). Therefore, in China, industrial sectors have implemented innovative activities to reduce energy consumption and industrial waste. However, the development of new products, as an important innovative activity in industrial production, increases the total energy consumption (27). Hence, R&D impact on industrial wastes as a comprehensive concept surrounding these innovative activities, needs to be studied further.

In the ancient era, Chinese rulers and thinkers always attached great importance to innovation. Whether it was personal self-cultivation or different methods of governing the country, the thinkers emphasized the need to rely on innovation to bring vitality. The Chinese classic, *The Book of Lord Shang*, stated that “*The three Dynasties have attained supremacy by different rites, and the five Lords Protector have attained their protector-ships by different laws*.”^2^ *The Great Learning* also stated, “*On the bathing-tub of T'ang, the following words were engraved: ‘If you can one day renovate yourself, do so from day to day. Yea, let there be daily renovation'*” (see footnote 1). Thus, in China, the concept of innovation was accepted a long time ago. At present, Chinese policy makers directly focus on the nexus between innovative activities and the current economy in practice. As an investment in innovation systems, R&D is considered to be the main indicator, to measure innovative activities and the innovative capacity of the local economy in China (28). With respect to market reforms in China, enterprises have become the main body of R&D investment and can thus, achieve significant positive growth from innovative activities (29). In China, the industrial sector, especially the manufacturing industry, emits large amounts of pollutants and consumes great amounts of energy; therefore, the dual goals of economic growth and environmental protection can be achieved by promoting the green transformation of the sector, along with technology innovation (30). To obtain profit, industrial producers need to ease the distortions of production by actively treating pollutant emissions under the strict environmental regulations (31). In the new era of China, policy makers adopt a new vision for development, i.e., *the vision of innovative, coordinated, green, and open development that is for everyone*. The vision of innovative and green developments indicates that R&D activities can help the country achieve industrial waste reduction in the production phase. In China, green product innovation can be promoted by implementing R&D tax incentives (32). The existing studies analyze how R&D activities surrounding waste treatment impact industrial wastewater, waste gases, and solid wastes separately (33–36). Whether an enterprise implements R&D innovation or not, and the amount of resources invested in these innovative activities, primarily depend on the profitability of the market (37, 38). In other words, R&D activities are implemented based on the market behavior and business decision of industrial producers. Several pollutants, including those from industrial wastes, are naturally emitted in the production phase, i.e., this is a natural process (39). As a result, the mechanism of a market behavior on the R&D impact on processes such as the emissions of industrial wastes must contain other factors, such as some environmental regulations and environmental protection laws (40). Additionally, environmental regulations regarding freshwater and atmospheric environments and solid waste can be applied at different intensities at the same stage of development. To obtain maximum profit and meet environmental protection requirements, industrial producers may implement R&D, to reduce one, two, or three types of industrial wastewater, waste gases, and solid wastes, respectively. Air pollution can cause respiratory diseases, cardiorespiratory diseases, cancers, etc., and impact public health (41), particularly in winter in China, fine particulate matter threatens human health (42). Additionally, industrial wastewater significantly impacts public health (43), especially in some developing countries (44). Industrial solid wastes, especially hazardous industrial wastes inevitably generated and affected human health (45). From the perspective of public health, industrial waste reduction from innovative activities will have a greater role in the process of development. In this study, the author analyzed the green effects of R&D on industrial waste reduction and determined their relations empirically, based on the data of China. By distinguishing the impacts of R&D on different industrial wastes, this paper was able to provide important policy recommendations, by revealing how environmental regulations can change industrial production.

Under the condition of maintaining public health, further elaborate the nexus between environmental quality and policies/regulations, and propose some new visions of innovative development to environment that could contribute some potential benefits to the wider public in the world. For the development of economy and society, public health becomes an important indicator to evaluate the status of development. Since industrial wastes negatively impact public health and innovative and green development probably reduce wastes and maintain growth, this paper is of great significance and interesting value in providing some policy recommendations. Notably, this paper also studied the response of industrial producers to emission limitations, in line with implementation of innovative activities. The author concludes that the coordination of green and innovative developments is a viable method for achieving sustainable development in the future. Industrial producers will not make decisions surrounding innovative inputs based on the objective of public health, although the existing studies have demonstrated that there was a nexus between R&D and industrial wastes in production, and maintaining public health needs to keep the environment clean. Rigorously analyzing the green effect of R&D on industrial wastes reduction could advance the current knowledge in the fields of public health and environmental protection under the vision of innovative and green development. Academic analysis surrounding the sustainable issues, including green and innovative development, and public health, does not limit to a specific region or industrial sector; deeply studying these issues nationally or globally could also advance the significance of this work. Under the influence of economic globalization and global environmental crisis, clarifying the heterogeneous green effects of R&D on industrial wastes in theoretical and empirical analysis could provide recommendations and research perspectives to analyze some environmental problems at the global level.

## Analysis

Under the competitive structure of the market, the managers of enterprises make decisions for R&D based on profit maximization. Many factors surrounding market operation could influence their decision, e.g., producers are more likely to implement R&D in an open economy than in a closed economy, because in an open economy, cost reduction through technological progress is relatively large (46). In the past, as they did not consider and control the external effects of production, industrial producers ignored the fact that R&D activities could reduce pollutant emissions. However, decisions related to R&D activities mainly depended on market operation, which often influenced the production process; this affected the pollutant emissions spontaneously. For example, in cases where policy makers do not implement any environmental regulations, producers will engage in R&D to increase productivity and decrease the input per unit output; this will reduce the emissions, while the output remains unchanged. In China, technological progress negatively impacts energy intensity (47). As energy consumption is the main reason for some pollutant emissions, technological progress is crucial for explaining the decrease in the pollutant intensity (48). Policy makers should be aware that the effect of R&D on pollutant emissions reduction is not due to the incentives of environmental regulations. Additionally, technological progress by implementing R&D activities can increase the total pollutant emissions, owing to the increase in the total output, even though the energy and pollutant intensities decline when the economy becomes more productive (49). R&D activities that reduce pollutant emissions may completely rely on market mechanisms, and in some scenarios, they cannot be regarded as green effects from public regulations. R&D innovation and pollutant emissions reduction indicate that the vision of green and innovative developments could be partially achieved by allocating resources effectively, in line with the market mechanism.

In China, the industrial sector consumes large amounts of energy and produces large amounts of waste in the production phase; therefore, many R&D innovation projects have been implemented in the sector. Policy makers need to control and constrain the behaviors of industrial producers, especially those regarding the emission of industrial wastes, based on the externality theory. In “*the concept that clear waters and green mountains are invaluable assets*” in China, environmental authorities formulated a series of strict environmental protection policies to prevent damage to the ecological environment. Industrial producers in China earn enormous profits; according to the *Statistical Bulletin on National Economic and Social Development of the People's Republic of China in* 2021^3^, the profit of industrial enterprises above designated size in 2021 was 8,709.2 billion yuan. Notably, industrial producers made most decisions in a profitable market environment in the economy; however, they should also consider their social responsibilities and ensure innovative and green developments. As a market behavior of positive social externality, policy makers always support industrial producers to implement R&D activities, to achieve innovative development. However, the development of new products that have high energy consumption and large pollutant emissions may be limited in practice. To protect the environment and save fossil energy, the development of new technology often entails technologies that are cleaner and green. Fiscal policies and associated financial implementations should focus on supporting the R&D activities that promote clean and green technologies (50). It is difficult to directly use environmental regulations to decide and regulate specific innovation behaviors of producers. In economic operation and environmental protection, policy makers often allow producers to emit certain amounts of pollutants and waste. Hence, producers could make any decision surrounding R&D activities in accordance with the relatively fixed environmental standards. In other words, it is difficult for policy makers to directly implement green characteristics and criteria that define the boundaries of R&D activities in industrial production.

Industrial wastes, including wastewater, waste gases, and solid wastes, are the by-products of the production phase. Generally, producers are aware of the discharges and emissions of associated pollutants. As industrial waste does not have any monetary value in the free market, producers generally do not measure the quantity of these emissions, if policy makers do not implement any restrictions on pollutant emissions during operation. To achieve green development and environmental sustainability, policy makers often set a maximum limit for the total pollutant emissions in industrial production. To optimize environmental regulations, it is particularly important to accurately measure the pollutant emissions in industrial production. The *Ministry of Ecology and Environment* of P.R.C., which is the authority in charge of protecting the environmental quality at the central government level in the state, monitors and regulates the behavior of pollutant emissions in industrial production. To date, the authority has implemented several environmental regulations, based on the relevant laws that limit industrial wastes, and requires industrial producers to monitor their own pollution discharge as well. In other words, it is the legal responsibility and obligation of industrial producers to measure their industrial waste in practice^4^ A few innovative activities, such as measuring the quantity of pollution, generally receive great support from policy makers. Developing technology to measure pollution could also be regarded as one of green characteristics of R&D activities in industrial operations. To release the distortion of production under the environmental regulations with maximum pollutant emissions, industrial producers implement R&D activities and green investments to treat pollution (51, 52). Although the R&D activity of measuring and treating industrial wastes directly meets the vision of green development, it is important to clarify that the green and indirect effects of the R&D on industrial waste reduction have significant theoretical value and are crucial for policy recommendations. However, producers mainly make decisions pertaining to R&D activities based on the market conditions.

For the society facing given sustainability-related challenges in many countries, public health should become an important objective along with sustainable development. The existing studies surrounding sustainability mainly focused on what factors could impact sustainable development, such as pollutants reduction, energy saving, etc. Sustainable Development Goals (SDGs) provide a feasible path for future development in the world; clarifying the green effects of R&D activities on industrial wastes reduction could further the knowledge of sustainability. Especially, taking the maintenance of public health as the policy objective to analyze the green effects of innovative activities could provide some significant recommendations to solve many associated sustainable challenges in the process of development. For example, renewable energy consumption helped to provide a pattern of production and consumption, which is free from environmental degradation under the 2030 United Nations SDGs (53). The environment, crucial for human survival, is a comprehensive system that contains many sections, e.g., the atmospheric, water, soil, geological, and biological environments. Different industrial wastes impact different environments, e.g., industrial waste gases mainly damage the atmospheric environment, and industrial wastewater degrades the quality of the water environment. Policy makers have developed different degrees of environmental regulations, to protect the associated environment. For example, Beijing, the capital of China, was one of the most polluted cities in the world; however, the quality of atmosphere improved significantly after policy makers implemented strict regulations (54). To emphasize the importance of public health, some associated authorities monitor industrial wastes and analyze its impacts on peoples' physiology, paying special attention to respiratory diseases (55). Additionally, the generation of hazardous wastes in industrial production often requires the strictest supervision, because these wastes can threaten public health. People's expectation of a good life mainly consists of good public health and a clean environment. As the impacts of different industrial wastes on public health vary in practice, environmental regulations are often formulated with heterogeneous limits for treating industrial wastewater, waste gases, and solid wastes. In general, industrial producers decide on specific R&D activities, while considering the different limits of different wastes. Tang (27) indicated that the development of new products had gasoline-saving characteristics and can thus, be beneficial to the economy of the country; crude oil in China is relatively scarce compared to other energy reserves. Industrial R&D activities are impacted by several external factors, such as energy status and public environmental regulations. Sustainable development and associated social and economic issues become the main contents in the previous work. And analyzing the green effect of R&D on industrial wastes reduction could advance and further these results, especially developing the theoretical framework of innovative development and green development could provide an important perspective to discuss the mechanism of environmental regulations formulation and public health maintenance.

Under the condition that environmental regulations have heterogeneous limits on different industrial wastes, the green effects of R&D should have specific characteristics. For example, if policy makers do not set any pollutant limits on the water environment, the producers' R&D activities may not focus on the green effects of industrial wastewater reduction, which could help these entities to obtain more profit in the competitive market but may impact the environment negatively. Instead, if authorities implemented strict regulations to protect atmospheric environment, producers would be willing to engage in R&D activities that promote the reduction of industrial waste gases. Directly researching the nexus between environmental policies and industrial wastes emissions may not be possible to discover the potential green environmental protection effect of the producer's innovative activities. As technological innovations in this study, empirically analyzing the heterogeneous effects of R&D on different industrial wastes reduction could reveal the production incentives of environmental policies and public regulations surrounding sustainable development. As a result, the novelty of this study and some new findings are mainly reflected by the mechanism of introducing policy recommendations in practice. To achieve sustainable development, formulating and implementing some strict environmental policies becomes the main public selection in many economies; however, the heterogeneous green effects of innovative activities in industrial production indicate that environmental policies for reducing industrial wastes could be automatically achieved along with developing rightly and innovatively in operation. At the theoretical level, rigorously analyzing the green effects of R&D on industrial wastes reduction could provide some new methods to study the relations among innovation, environment, and growth in further theoretical analysis. Sustainable development contains public health, innovative development, and green development, respectively. A framework containing these associated issues in this work is helpful to theoretically study the feasible path of sustainable development in the future.

## General description of industrial wastes emissions and R&D in China

### Industrial wastes emissions in China

Before empirical analysis, this paper first revealed the trend of the changes in industrial wastes and R&D activities in the industrial sector of China. For industrial wastes, the author acquired the data from the *China Statistical Yearbook on Environment*, known to contain useful and accurate information. Industrial wastes, including industrial wastewater, waste gases, and solid wastes, were recorded in the yearbook. More specifically, the data on raw materials contained information about the discharged chemical oxygen demand (COD) and ammonia nitrogen (included in the items under industrial wastewater), industrial sulfur dioxide, nitrogen oxides, and particulate matter emissions (included in the items under industrial waste gases), and generated common industrial solid wastes and hazardous wastes (included in the items under industrial solid wastes). Additionally, the data of industrial wastes after 2016 was not comparable to that of the previous year, based on the brief introduction from the publisher. Therefore, in this study, the author used the data for 2016–2020, to conduct an empirical test.

**Figure 1:**
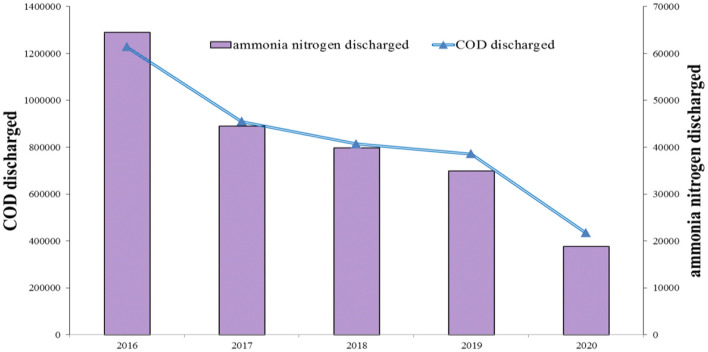
Discharge of industrial wastewater in China during 2016–2020 (ton). Source: The author obtained the information from the *China Statistical Yearbook on Environment*.

The discharge of industrial wastewater, including the COD and ammonia nitrogen discharged by the industrial sector in China during 2016–2020 is depicted in Figure 1. The data surrounding industrial wastes contain the information on the *professional and support activities for agriculture, forestry, animal husbandry, and fishery*, which is generally not included in the classification of the industrial sector of China^5^. To find the trend of the change in the total pollutant emissions, a general description surrounding industrial wastes contains the data in the extra sector. Based on Figure 1, the discharge of COD and ammonia nitrogen in industrial sector reduced in China during 2016–2020. Furthermore, the discharge of industrial wastewater in 2020 significantly declined. The economic growth slowed down in 2020, due to the COVID-19 pandemic, which may have resulted in the reduction of industrial wastewater (56).

**Figure 2:**
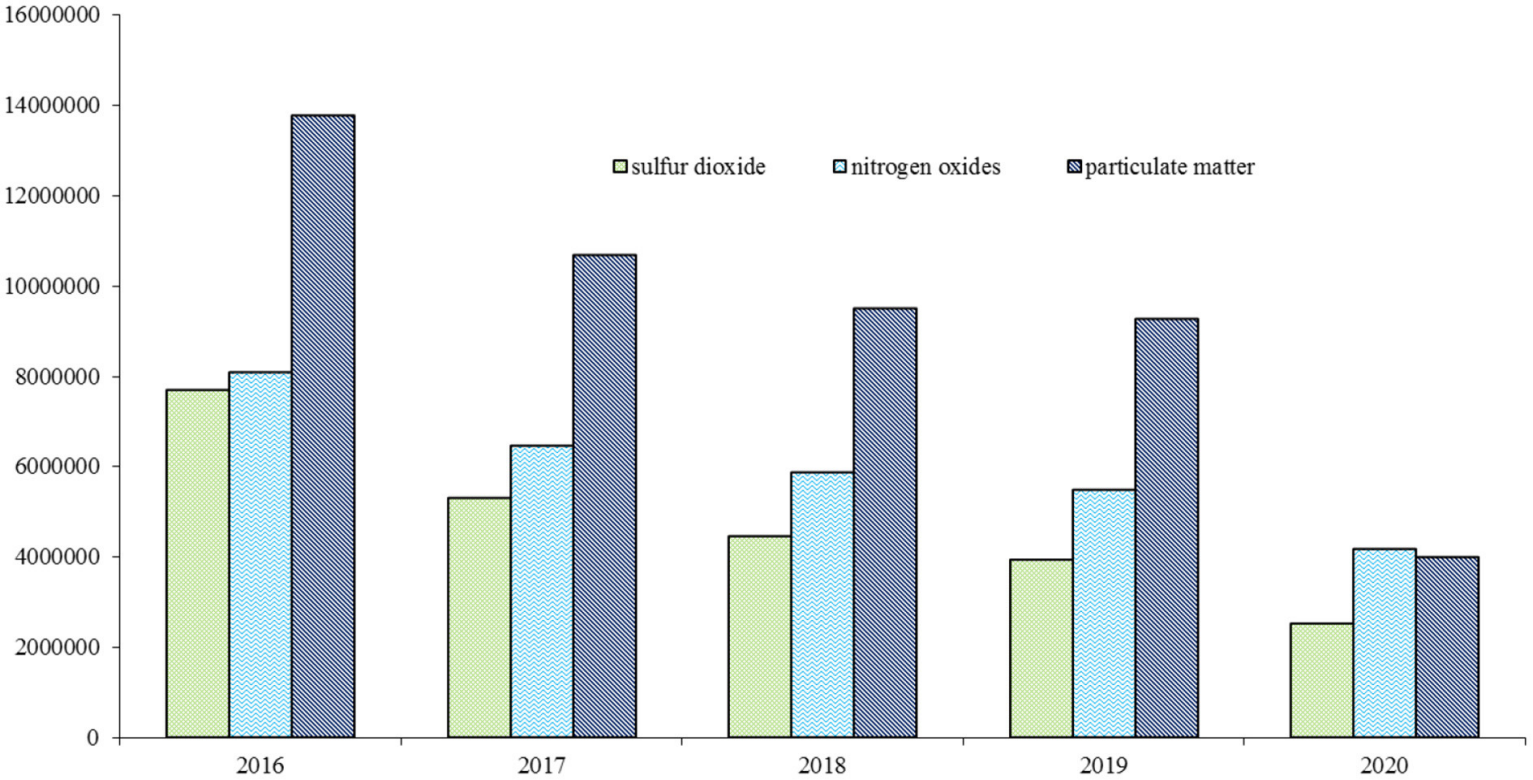
Emission of industrial wastes gases in China during 2016–2020 (ton). Source: The author obtained the information from the *China Statistical Yearbook on Environment*.

Figure 2 portrays the emissions of industrial waste gases, including sulfur dioxide, nitrogen oxides, and particulate matter, in China during 2016–2020. The reduction trend of the emissions of these three industrial waste gases was significant during this period. The changing trend of industrial waste gases was similar to that of industrial wastewater (Figures 1, 2). Notably, industrial particulate matter emissions portrayed the most significant decline. Industrial particulate matter emissions far exceeded the industrial sulfur dioxide and nitrogen oxides emissions in 2016; however, in 2020, they were lower than the industrial nitrogen oxides emissions. In China, people focus on the nexus between particulate matter and public health; in recent years, Chinese cities have reported high PM2.5 concentrations (57). In most cases, policy makers have implemented more stringent control policies for industrial particulate matter emissions in response to public concerns regarding their health and PM2.5 levels. As a result, the reduction in the industrial particulate matter emissions in China is significant; this indicates that environmental regulations for different waste products should specify different limits.

**Figure 3:**
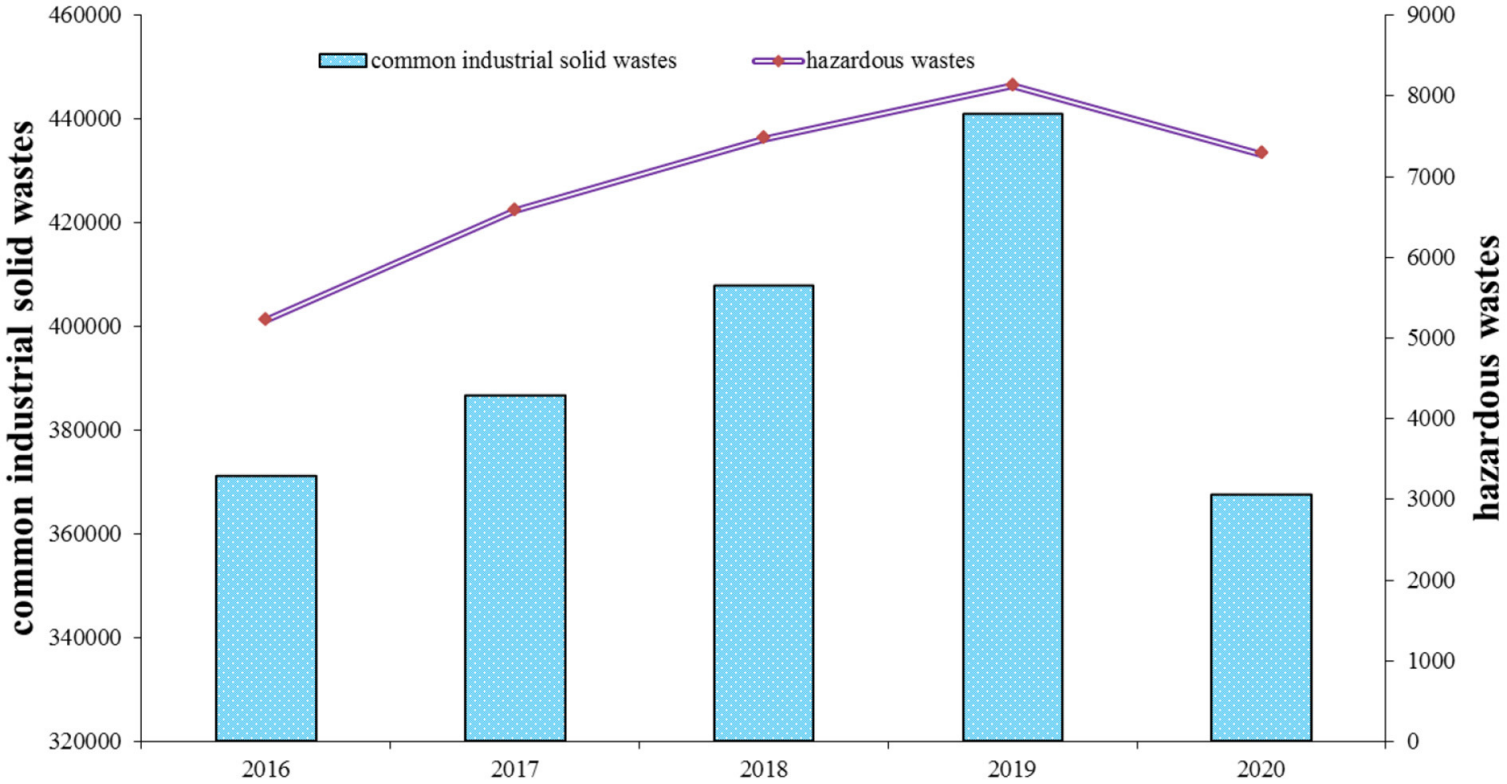
Generation of industrial solid wastes in China during 2016–2020 (10000 tons). Source: The author obtained the information from the *China Statistical Yearbook on Environment*.

The generation of common industrial solid and hazardous wastes in China during 2016–2020 is illustrated in Figure 3. Unlike the reduction of industrial wastewater and waste gases in the same period, common industrial solid and hazardous wastes increased gradually during 2016–2019 and significantly declined in 2020. Due to the COVID-19 pandemic, 2020 was the most unusual year for the world, including China. Notably, there was a significant reduction in the total municipal solid waste in China in 2020 (58), in line with the decrease in industrial solid wastes. Due to environmental regulations, industrial producers want to reduce the solid wastes in the production phase. However, it is not easy to significantly reduce industrial solid wastes by only implementing innovative activities. Macroeconomic situation is more likely to impact production process and change production pattern of total industrial solid wastes.

During 2016–2020, there was a significant reduction in the industrial wastewater and waste gases in the industrial sector of China, but industrial solid wastes in the sector increased during 2016–2019, followed by a significant reduction in 2020. The general description surrounding the changing trend of different industrial wastes could preliminarily reveal some characteristics of the industrial production processes and environmental regulations in China.

### R&D activities in the industrial sector of China

With the vision of innovative development, policy makers in China focus on the optimal use of innovation to drive economic and social development. The Chinese economy could not buy or request core technologies in some key fields from other economies of the world. To implement innovative activities and allocate the elements of innovation in the market environment, the policy makers in China clearly define that enterprises are the main body and players for innovation (59); industrial enterprises are also the main entities that initiate innovative activities (27). The amounts of industrial wastes are significantly large in China; there is a nexus between the innovative activities in this sector and the environmental regulations (especially those that entail controlling pollutant emissions) imposed by policy makers.

**Figure 4:**
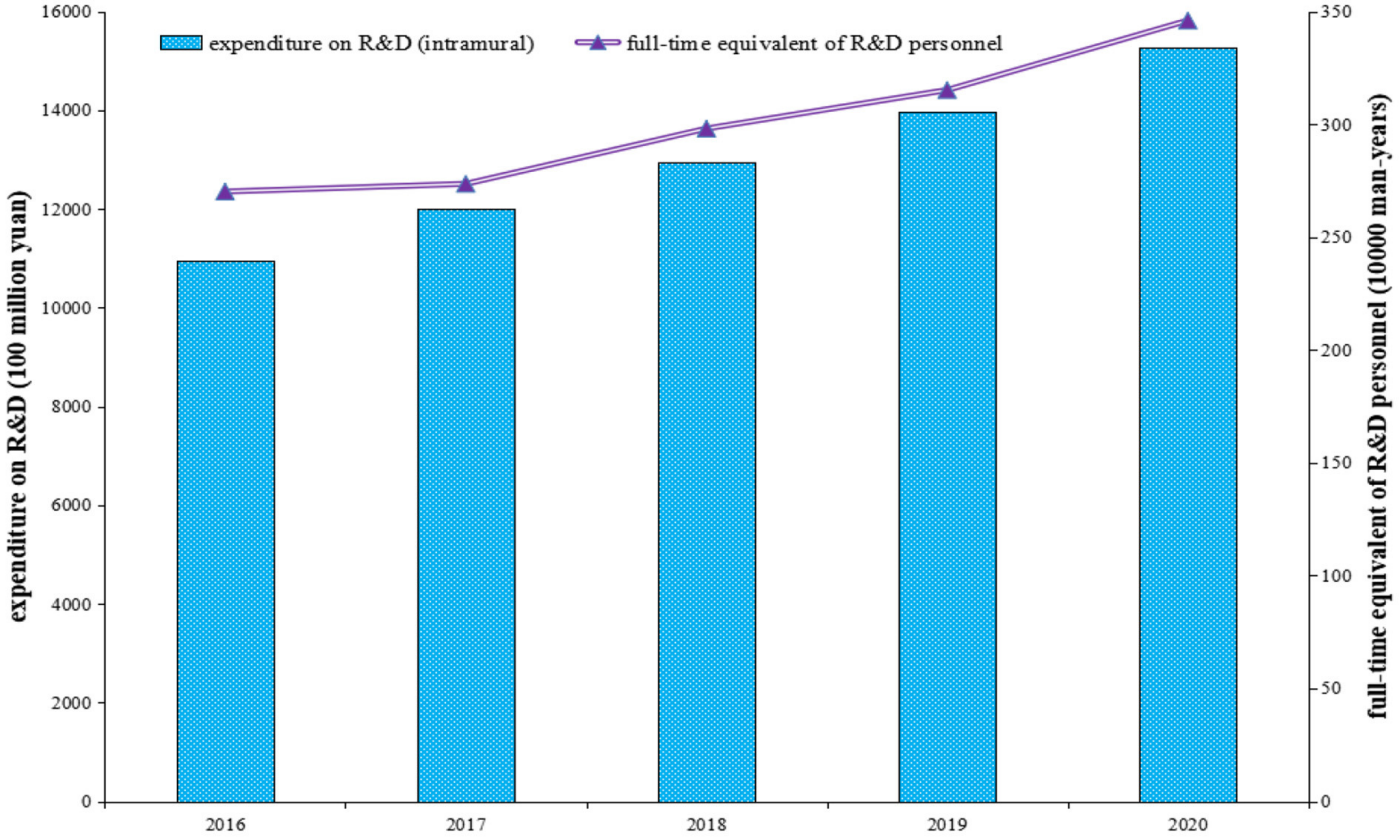
Research and development (RandD) activities in the industrial sector in China during 2016–2020. Source: The author obtained the information from the *China Statistical Yearbook on Science and Technology*.

The *China Statistical Yearbook on Science and Technology* contains useful data regarding the status of science and technology in the country, including R&D. During 2016–2020, the main measurements of R&D activities for the industrial sector of China, including the full-time equivalent of R&D personnel and expenditure on R&D (intramural), are depicted in Figure 4. Overall, the human and monetary resources surrounding the innovative activities implemented in the industrial sector of China increased significantly. For example, the total expenditure on R&D (intramural) in the industrial sector in 2020 was 15,271.3 (100 million yuan).

**Figure 5:**
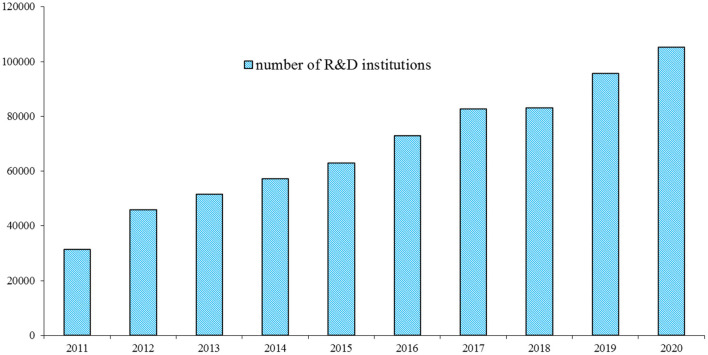
Number of research and development (RandD) institutions located in the industrial sector in China during 2011–2020 (unit). Source: The author obtained the information from the *China Statistical Yearbook on Science and Technology*.

Additionally, in China, industrial producers are allowed to implement R&D activities directly. Figure 5 portrays the number of R&D institutions located in the industrial sector in China during 2011–2020. Over time, the number of R&D institutions located in the industrial sector increased. Notably, several enterprises constructed independent R&D institutions; this indicated that the managers of these enterprises paid more attention to innovation in the mechanism of the market. In the industrial sector of China, the intramural expenditure on R&D was more than the external expenditure on R&D; this may be because the industrial enterprises could directly implement R&D activities in their own innovative institutions. Therefore, in this study, the author used the data on the intramural expenditure on R&D, rather than the external expenditure, to measure the monetary input of R&D, using an empirical test.

The managers of the Chinese industrial sector pay close attention to innovative activities at the micro level, which could make valuable contributions to high-quality development at the macro level, in line with the vision of innovative development. If these R&D inputs could also have green effects on industrial wastes reduction, the vision of green development could also be achieved simultaneously. In the following section, the author has detailed the empirical tests applied in this study, to determine the impacts of R&D activities in the industrial sector on industrial waste discharge.

## Empirical analysis

### Data materials

In China, “*mining*,” “*manufacturing*,” and “*production and supply of electricity, gas, and water*” are the three main components of the industrial sector. Additionally, these three sectors also contain 41 smaller segments (27). As described above, the data surrounding industrial wastes in the *China Statistical Yearbook on Environment* contains 42 sectors, along with an added sector “*professional and support activities for agriculture, forestry, animal husbandry, and fishery*.” To construct a balanced panel data using other materials, the author did not consider the latter sector.

The data surrounding the industrial R&D activities from the *China Statistical Yearbook on Science and Technology* did not include three sectors: “*professional and support activities for mining*,” “*mining of other ores*,” and “*utilization of waste resource*.” Hence, the author did not consider the data of these three sectors from other yearbooks, to build a strongly balanced panel data. Additionally, the statistical authority renamed some industrial sectors in the period, but it did not seriously influence empirical analysis (27). Thus, the author used the data from 38 sectors in the industrial sector and considered a period of 5 years (2016–2020) for empirical analysis, i.e., a short panel data. The structure of the short panel data helped the author to obtain reliable results from fixed effect regressions.

Finally, the author used the *China Statistical Yearbook* to acquire the data of the industrial performance in operations. According to the nexus between the industrial wastes and energy consumption in the production phase, the variability in energy usage was considered in the regression. The data on energy consumption in industrial sector was acquired from the *China Energy Statistical Yearbook*. The four yearbooks used for the empirical analysis were published by the *China Statistical Press* and compiled by official statistical authorities. The author assumed that the data were accurate, based on the authority of the sources. To validate the results in empirical analysis, ensuring the obtained data comes from authoritative raw materials will be important. Although the obtained data mainly from the Chinese industrial sectors during 2016–2020, the empirical results could also provide some implications to implement public policies at the level of industry, economy, and wider society, respectively. First, empirically clarifying the green effects of R&D on industrial wastes emissions could guide industrial producers to alleviate the distortion of environmental regulation on production. Second, the empirical findings could provide policy recommendations for policy-makers to formulate an appropriate path of development with the vision of innovation and green in an economy. Finally, taking public health as an element in the empirical and policy analysis could help people focus on the clean environment and health in the wider society.

### Variables and method

The dependent variable, *Y*, mainly represented the quantity of some industrial wastes. According to the raw materials, the discharges of COD and ammonia nitrogen, emissions of sulfur dioxide, nitrogen oxides, particulate matter, and generation of common industrial solid and hazardous wastes were recorded by the industrial sectors; the information was used to build the dependent variables for the empirical tests. To conduct accurate empirical tests, the author considered the dependent variables using the logarithm of the recorded data (Table 1).

**Table 1:** Dependent variables in empirical analysis.

**Variables**	**Measurement**	**Item**
*Ln_COD*	*Ln_COD* = ln(*COD*), where *COD* was the discharge of COD	Industrial wastewater
*Ln_AN*	*Ln_AN* = ln(*AN*), where *AN* was the discharge of ammonia nitrogen	Industrial wastewater
*Ln_SO_2_*	*Ln_SO_2_* = ln(*SO_2_*), where *SO_2_* was the emission of sulfur dioxide	Industrial waste gas
*Ln_NOx*	*Ln_NOx* = ln(*NOx*), where *NOx* was the emission of nitrogen oxides	Industrial waste gas
*Ln_PM*	*Ln_PM* = ln(*PM*), where *PM* was the emission of particulate matter	Industrial waste gas
*Ln_CISW*	*Ln_CISW* = ln(*CISW*), where *CISW* was the generation of common industrial solid wastes	Industrial solid wastes
*Ln_HW*	*Ln_HW* = ln(*HW*), where *HW* was the generation of hazardous wastes	Industrial solid wastes

The independent variable, *X*, for the R&D activities denotes the intramural expenditure on R&D and the full-time equivalent of R&D personnel. *Ln*_*R*_&*D* and *Ln*_*R*_&*D*_*P*_ represent the logarithm of the intramural expenditure on R&D and the full-time equivalent of R&D personnel, respectively. The author established the monetary resources, based on the intramural expenditure on R&D and human resources, based on the full-time equivalent of R&D personnel being the independent variables, to investigate the robustness of the empirical results. Additionally, the output of innovative activities, including patent, also impact the industrial wastes. This paper mainly measures the innovative activities by R&D input to empirically test. In the further studies, empirically testing the nexus between patent and industrial wastes will provide other perspectives in the field.

Finally, the author introduced the control variables for the regression equations, based on the characteristics of the raw materials and objectives of the empirical analysis, as follows: (1) Energy consumption portrayed a significant correlation with industrial wastes and impacted the producers' decisions on the R&D activities. *Ln*_*energy*_= ln(energy) could be used in empirical tests, where [[Inline Image]] represented the total energy consumption (standard quantity) in the industrial sectors. (2) The business revenue represented the status of industrial operation and scale of the sector. More production with more pollutant emissions is a common pattern in industrial production. Big enterprises and sectors often undertake large R&D projects and input huge innovative resources. Notably, the author observed that, during 2016–2020, raw materials recorded the business revenue or revenue from the principal business. A variable *Ln*_*R*_ = *ln*(*revenue*) could be built by using the logarithm of (*revenue*), where (*revenue*) is the value of business revenue or the revenue from the principal businesses in the industrial sector. (3) The total assets and the annual average number of employees in these industrial sectors indicate the input factors in production, which may influence the discharge of industrial wastes and the associated innovative decisions. The author considered two variables, namely, *Ln*_*K*_ = *ln*(*K*) and *Ln*_*L*_ = *ln*(*L*), for empirical test, where *K* and *L* represent the total assets and the annual average number of employees recorded in raw materials, respectively. (4) Producers with more profit could reduce industrial waste by implementing clean technologies and green practices. Additionally, positive or negative profits also influenced the R&D decision during the production phase. Raw materials recorded total profits in these industrial sectors; some sectors had a positive profit in some years, whereas others experienced losses. To use the logarithm of total profits, with positive and negative value, the author considered the variable, *Ln*_*profit*_ = sgn(*profit*)ln[sgn(*profit*)*profit*], where *profit* represented the total profits in the industrial sector; the symbolic function sgn(.) was used to determine the positive or negative value after logarithm transformation. The total profit was measured by 100 million yuan, with not a single sector having a total profit under the region [−1, 1]. Thus, applying the logarithm of the total profits could ensure accurate results.

After defining the variables, the author tested the following equation, using fixed effect regression; the strongly balanced panel data could reveal the influence of R&D activities on the industrial wastes in China. The equations used for the empirical analysis are explained below:


$$\begin{array}{l} Y_{it}=C+\beta_{1}X_{it}+\beta_{2}Ln_{energy_{it}}+\beta_{3}Ln_{R_{it}}+\beta_{4}Ln_{K_{it}}+\beta_{5}Ln_{L_{it}}\\ \;+\beta_{6}Ln_{Profit_{it}}+\varepsilon_{it} \end{array}$$

where *Y*_*it*_ represents *Ln*_*CO*_*D*__*it*__, *Ln*_*A*_*N*__*it*__, *Ln*_*No*_*x*__*it*__, *Ln*_*P*_*M*__*it*__, *Ln*_*CIS*_*W*__*it*__, and *Ln*_*H*_*W*__*it*__. *X*_*it*_ represents *Ln*_*R&*_*D*__*E*_*it*___, and *Ln*_*R&*_*D*__*P*_*it*___. The subscript *Ln*_*CIS*_*W*__*it*__ and *Ln*_*H*_*W*__*it*__ in the equation represent the group/observation (industrial sector) *Ln*_*R&*_*D*__*P*_*it*___ in the year *t* of the associated variables.

## Results

First, the author recorded the results of the influence of R&D activities on the industrial wastewater in China, using the empirical analysis method explained in section “Variables and method”. The results are illustrated in Table 2. The results of the partial effect of R&D on industrial wastewater and its statistical significance were robust. Furthermore, the fact that the industrial sectors implemented more R&D activities could justify the reduction in the discharge of COD in the production phase. The negative effects of R&D on the discharge of ammonia nitrogen were not statistically significant.

**Table 2:** Nexus between research and development (RandD) activities and industrial wastewater.

**Variables**	***Ln_COD***	***Ln_COD***	***Ln_AN***	***Ln_AN***
*_cons*	22.7133*** (4.1264)	21.9600*** (4.1996)	19.1769*** (5.3828)	17.1314*** (5.4330)
*Ln_RandD_E*	−1.0096*** (0.2792)		−0.5762 (0.3642)	
*Ln_RandD_P*		−0.8216*** (0.2938)		−0.0182 (0.3801)
*Ln_energy*	−0.4342 (0.3856)	−0.4701 (0.3944)	−1.0458** (0.5031)	−1.2089** (0.5102)
*Ln_R*	0.5015 (0.4384)	0.0149 (0.4603)	0.4500 (0.5719)	0.3587 (0.5954)
*Ln_K*	−0.7442 (0.4726)	−1.0021** (0.4720)	−0.9066 (0.6165)	−1.4687** (0.6106)
*Ln_L*	1.2297** (0.4911)	1.7982*** (0.4671)	1.4266** (0.6406)	1.7870*** (0.6043)
*Ln_profit*	0.0020 (0.0403)	−0.0300 (0.0420)	0.0013 (0.0526)	−0.0027 (0.0543)
*R* ^2^	0.3898	0.3690	0.2574	0.2447
*F*-test	Prob > *F* = 0.0000	Prob > *F* = 0.0000	Prob > *F* = 0.0000	Prob > *F* = 0.0000
Number of obs	190	190	190	190

Symbols ^***^, ^**^, and ^*^ represent statistically significant at the level of 1%, 5%, and 10%, respectively. Standard errors are recorded in the brackets.

Second, Table 3 portrays the empirical results of the partial effects of R&D activities on industrial waste gases. Based on the results in Table 3, the partial effects and statistical significance of R&D, in terms of the monetary and human resources, on industrial waste gases were robust. For the three industrial waste gases, namely sulfur dioxide, nitrogen oxides, and particulate matter, the industrial sectors implemented R&D activities that could significantly reduce their emissions in practice. In other words, the R&D activities in the industrial sector of China had significant green effects on industrial waste gas reduction. The problem of global climate change is mainly produced by the source of human-induced changes in atmospheric composition (60); therefore, human behavior not only causes global climate change but also responds and adapts to it (61). To evaluate global climate scenarios, accurately calculating regional energy usage, industrial production and greenhouse gas are very important (62). The reduction effect of innovative activities on industrial waste gases is possible to provide a feasible method to reduce the greenhouse gases in practice.

**Table 3:** Nexus between research and development (RandD) activities and industrial waste gases.

**Variables**	***Ln_SO_2_***	***Ln_SO_2_***	***Ln_NOx***	***Ln_NOx***	***Ln_PM***	***Ln_PM***
*_cons*	25.6816*** (6.1899)	26.6196*** (6.1292)	28.4558*** (4.5618)	28.8908*** (4.5227)	35.0772*** (7.1858)	36.5429*** (7.0669)
*Ln_RandD_E*	−1.0006** (0.4188)		−1.2155*** (0.3087)		−1.3777*** (0.4862)	
*Ln_RandD_P*		−1.2842*** (0.4288)		−1.3635*** (0.3164)		−1.8168*** (0.4944)
*Ln_energy*	0.1358 (0.5785)	0.2489 (0.5755)	0.1704 (0.4263)	0.2456 (0.4247)	0.1686 (0.6715)	0.3396 (0.6636)
*Ln_R*	0.8374 (0.6577)	0.1609 (0.6717)	1.3139*** (0.4847)	0.5737 (0.4957)	1.1591 (0.7635)	0.2075 (0.7745)
*Ln_K*	−2.3162*** (0.7089)	−2.1392*** (0.6889)	−2.2512*** (0.5225)	−2.2171*** (0.5083)	−2.6744*** (0.8230)	−2.3860*** (0.7942)
*Ln_L*	2.2386*** (0.7367)	2.7646*** (0.6817)	1.2397** (0.5429)	1.8943*** (0.5030)	1.7510** (0.8552)	2.4713*** (0.7860)
*Ln_profit*	0.0057 (0.0605)	−0.0409 (0.0613)	0.0016 (0.0446)	−0.0488 (0.0452)	−0.0139 (0.0702)	−0.0796 (0.0707)
*R* ^2^	0.3913	0.4041	0.5116	0.5207	0.3671	0.3888
*F*-test	Prob > *F* = 0.0000	Prob > *F* = 0.0000	Prob > *F* = 0.0000	Prob > *F* = 0.0000	Prob > *F* = 0.0000	Prob > *F* = 0.0000
Number of obs	190	190	190	190	190	190

Symbols ***, **, and * represent statistically significant at the level of 1%, 5%, and 10%, respectively. Standard errors are recorded in the brackets.

Finally, the effect of the intramural expenditure on R&D and the full-time equivalent of R&D personnel on industrial solid wastes was empirically tested (Table 4). Statistically, in enterprises that are in operation, R&D activities have no significant effects on industrial solid wastes. The equation could not analyze the generation of hazardous wastes, the main reason being that the industrial solid waste was generated as a part of the production process, and the market variables for the R&D activities could not be easily influenced in the production phase. To improve the circular economy of industrial waste, developing the capacity of recycling industrial waste will be a favorable option to sustain industrial development in the future (63). Since the green effect of R&D on industrial solid wastes reduction is not significant statistically, recycling industrial solid wastes more fully become a main objective while implementing environmental policies for managing these solid wastes (64). The ratio of industrial solid wastes utilized should attract policy-makers' attention in practice.

**Table 4:** Nexus between research and development (RandD) activities and industrial solid wastes.

**Variables**	***Ln_CISW***	***Ln_CISW***	***Ln_HW***	***Ln_HW***
*_cons*	7.9573* (4.2155)	8.7073** (4.1972)	−12.3809* (6.3522)	−11.7903* (6.3601)
*Ln_RandD_E*	−0.2034 (0.2852)		0.1557 (0.4298)	
*Ln_RandD_P*		−0.4171 (0.2936)		−0.0056 (0.4450)
*Ln_energy*	1.0317*** (0.3940)	1.1040*** (0.3941)	−0.1438 (0.5937)	−0.0964 (0.5972)
*Ln_R*	0.0060 (0.4479)	−0.1961 (0.4600)	0.4840 (0.6749)	0.5044 (0.6970)
*Ln_K*	−0.9878** (0.4828)	−0.8082* (0.4717)	0.9762 (0.7275)	1.1378 (0.7148)
*Ln_L*	0.6117 (0.5017)	0.7062 (0.4668)	0.0045 (0.7560)	−0.0937 (0.7074)
*Ln_profit*	0.0539 (0.0412)	0.0395 (0.0420)	−0.0196 (0.0621)	−0.0188 (0.0636)
*R* ^2^	0.1156	0.1246	0.0556	0.0547
*F*-test	Prob > *F* = 0.0058	Prob > *F* = 0.0031	Prob > *F* = 0.2062	Prob > *F* = 0.2150
Number of obs	190	190	190	190

Symbols ***, **, and * represent statistically significant at the level of 1%, 5%, and 10%, respectively. Standard errors are recorded in the brackets.

All in all, the green effect of R&D on industrial waste gas reduction was significant. For industrial wastewater, R&D had a significant green effect on COD reduction; however, the effect on ammonia nitrogen was not significant. As the industrial sectors generated solid wastes that mainly depended on natural processes, the R&D activities had no significant impact on the common industrial solid and hazardous wastes. Based on these empirical findings, the author proposed important policy recommendations, with the vision of innovative and green developments.

## Policy recommendations

In general, people prefer to live in a clean environment and lead a healthy life. Policies that promote a sustained lifestyle should be established in economic operations (65). However, several pollutants threaten public health; therefore, policy makers in China have implemented strict environmental regulations to reduce industrial wastes in economic operations. Innovation and technological progress could drive economic growth and increase income; consequently, people with higher incomes will pay more attention to environmental quality and health. Based on historical experience, people mainly focus on economic growth, without considering the environment. In this era of poverty and low environmental quality, people do not even pay attention to their own health. Industrialization has brought great benefits to mankind; however, it has also caused serious problems, e.g., excessive energy consumption and environmental degradation. Some industrial wastes directly influence public health, e.g., high particulate matter levels can cause respiratory disorders. As the emission of industrial particulate matter accounts for the highest proportion of total emissions of particulate matter in China (9), the environmental regulations pertaining to the emissions of industrial particulate matter are strict. Due to market competition, an effective environmental policy should not impact the production seriously, otherwise, such a regulation will be difficult to implement persistently. Notably, innovative activities could ensure sustainable development, with high quality. Innovation in the industrial sector is often regarded as a viable method for energy saving and pollution reduction, which are the main goals of green development. According to empirical analysis, based on the panel data of China, the author observed that R&D activities had significant green effects on industrial waste gas reduction. Furthermore, in China, policy makers pay more attention to the protection of the atmospheric environment and implement strict regulations to limit the emissions of waste gases. In this study, the author considered R&D activities, as market behavior, in response to the environmental regulations pertaining to industrial waste gas reduction; industrial producers would prioritize the R&D projects that had the green characteristics of decreasing industrial waste gaseous emissions. Furthermore, environmental policies at the macro level could indirectly establish the green effect of R&D activities in the production phase at the micro level. Thus, innovative and green developments could be automatically achieved, based on the green effects of R&D on the industrial waste gas reduction in the industrial sector of China.

In general, an economy consumes more energy with increasing productivity (49). In such cases, technological progress will increase the total output and decline the energy and pollution intensities. However, the total energy consumption and total pollutant emissions will increase with increasing production; this is especially true for developing economies, such as China. At the micro level, a firm with higher productivity has more emissions. If the output in the firm remains unchanged, the pollutant emissions will decline with increasing productivity (66). The Chinese economy has experienced significant technological progress and an increase in energy consumption; however, in recent years, the discharge of industrial wastewater and emissions of industrial waste gas have been reduced with a significant increase in the R&D inputs in the industrial sector. The green effects of the R&D activities on COD discharge and all the reduction in the industrial waste gases have been statistically verified. Energy saving and pollution reduction are the goals of green development; therefore, the green effects of R&D, e.g., saving energy, especially fossil energy, should be given more attention in the future. Notably, R&D activities with technological progress often increase energy consumption, owing to the increase in production. The activities implemented by the industrial producers have several targets. Technological progress that is based on R&D activities is more likely to increase energy consumption and pollutant emissions. However, other goals of R&D activities may have significant green effects on pollution reduction.

In this study, R&D activities could not reduce the common industrial solid and hazardous wastes that are the two main types of industrial wastes. Notably, industrial solid wastes are away from public sight, even though public health is seriously threatened by hazardous wastes and the associated industrial solid wastes as well. Due to the lack of public supervision, environmental regulations on the reduction of industrial solid wastes may not be strict. People focus more on air pollution; thus, industrial producers implemented R&D activities that paid more attention to reducing industrial gaseous wastes with strict limits for atmospheric pollutant emissions. Furthermore, policy makers have formulated rigorous limits for the generation of industrial solid wastes, which will promote producers to implement innovative activities to reduce solid wastes and enhance the green effects of R&D. Additionally, some industrial waste residues are inevitably generated in the production phase. It is difficult to decrease the generation of industrial solid wastes that are generated as a part of the production process, thus the R&D input did not have significant effects on solid wastes.

The generation of industrial solid wastes is difficult to reduce, therefore, industrial producers actively treat these pollutants using clean technology to solve the associated environmental problems. R&D activities for treating pollution have green effects on pollutant emissions treatment. To achieve innovative development, Chinese policy makers support industrial producers to implement R&D activities through subsidies and tax reductions, such as preferential tax policies, e.g., by granting an extra tax deduction on the enterprises' R&D costs (67). The industrial sectors that implement more R&D activities could significantly decrease industrial waste gases and the discharge of COD in China. To achieve green development, policy makers mainly support innovative activities that entail the development of cleaner technologies. Enterprises implement R&D activities that are profit based, depending on the conditions of the competitive market. Therefore, fiscal policies with R&D subsidies should focus on the development of new cleaner technologies that promote energy saving, pollution reduction, and emissions treatment. Although the reduction of industrial wastewater and waste gas has lasted several years in the Chinese economy, their harm to public health and ecological environment should not be underestimated. For a clean environment, these pollutants need to be treated at the production phase itself. Hence, the R&D activities that promote cleaner technologies should have more support than the general R&D projects. These R&D activities do not possess any associated effect on production with the technological progress and development of new products; producers do not implement them for a long time. In other words, the profitability and operation in the competitive market is the main goal of R&D. To achieve innovative and green developments, general R&D activities in the Chinese industrial sectors should have significant green effects on the reduction of industrial wastes, including the three main types of industrial waste gases and one type of industrial wastewater. Furthermore, the green effects of R&D on these industrial wastes reduction should be expanded and industrial waste reduction should be the main goal of public policies. An economy could, based on the path of innovative and green developments, protect public health by decreasing pollutant emissions when the green effects of R&D on industrial wastes reduction are significant and industrial producers are willing to implement R&D activities in practice.

## Conclusions

Along with economic development and social progress, people pay attention to the quality of life. A clean environment, as an important essential element of a healthy life, has gradually become a public goal pursued by policy makers in several countries. Due to the serious threat to public health, the reduction of industrial waste emissions is important for smooth economic operation and industrial production. Policy makers could formulate strict environmental regulations to limit the emissions of pollutants, but this method may impact the economic development. For green development, industrial producers may implement R&D activities that could reduce industrial wastes and coordinate the public goal of economic development and environmental protection. In the case of market competitions, R&D activities of industrial producers are business behavior, i.e., are used to pursue larger profits in operation. Most pollutant emissions are only by-products in the production phase, generated by a natural process. Environmental policies with limits for pollutant emissions probably influence and distort the production in practice.

In China, the discharge of industrial wastewater and emissions of industrial waste gas reduced during 2016–2020, but generations of industrial solid wastes increased during 2016–2019, followed by a significant reduction in 2020. Industrial sectors have input an increasing amount of resources to implement R&D activities. The Chinese environmental regulations were implemented strictly in recent years, and industrial producers may implement some R&D activities with green effects, to reduce industrial wastes. In this study, the author empirically tested the green effects of R&D activities on industrial wastewater (including discharge of COD and ammonia nitrogen), industrial gaseous waste emissions (including emission of sulfur dioxide, nitrogen oxides, and particulate matter), and industrial solid wastes (including generation of common industrial solid wastes and hazardous wastes), and observed that the green effects of R&D on reduction of all three industrial waste gases were statistically significant. Chinese policy makers have to implement strict regulations to protect the atmospheric environment as people tend to monitor air quality in daily life, i.e., the limitation of industrial waste gas emission from environmental policies is clear. To ease the distortion of production from the limitation of emissions, industrial producers are more likely to implement innovative activities to reduce the industrial waste gases. Additionally, the green effect of R&D on the reduction of discharge of COD was also statistically significant, but the partial effect on the discharge of ammonia nitrogen was not statistically significant. The partial effect of R&D on the two types of industrial solid wastes reduction was not statistically significant as well. Clarifying the heterogeneous green effect of R&D on different industrial wastes reduction could provide important policy recommendations to develop sustainably under the vision of innovative and green development. For improving public health, the fiscal policies not only support general R&D activities, with green effects on industrial wastes reduction, but also pay more attention to the special R&D activities for pollution treatment through development of cleaner technologies. Notably, pollutant emissions generated in process of production will always pose risks and must be addressed to avoid serious threats to the health of people.

Public health, clean environment, innovative and green development, industrial wastes emissions and their reduction trend, etc., have become the worldwide issue and attracted a large number of researchers and policy-makers in many countries. This paper mainly analyzes the green effect of R&D on industrial wastes reduction and provides some policy recommendations to implement and formulate at the world level. As a result, this study could further worldwide knowledge and policy implications on these topics significantly. However, clarifying the green effects of R&D on different industrial wastes based on China's evidence solely is the main limitation and bottleneck of this work. For public health and its measurement indicator, regional data, including the expectation of life in province, city, etc., is the main material used in the associated empirical analysis. In this study, industrial wastes and R&D recorded at the level of industrial sectors could not directly assess the impact on public health; therefore, the nexus between public health and clean environment is only used as a priori condition in the section of empirical analysis and policy implications. According to the green effects of R&D on these industrial wastes in empirical analysis, further research direction by directly studying how R&D activities impact public health will be significant in the process of future sustainable development with the vision of innovative and green development in the world.

## Data availability statement

The original contributions presented in the study are included in the article/supplementary material, further inquiries can be directed to the corresponding author/s.

## Author contributions

The author confirms being the sole contributor of this work and has approved it for publication.

## Conflict of interest

The author declares that the research was conducted in the absence of any commercial or financial relationships that could be construed as a potential conflict of interest.

## Publisher's note

All claims expressed in this article are solely those of the authors and do not necessarily represent those of their affiliated organizations, or those of the publisher, the editors and the reviewers. Any product that may be evaluated in this article, or claim that may be made by its manufacturer, is not guaranteed or endorsed by the publisher.

## Footnotes

^1^The English version was published by Liaoning People's Publishing House in 2017, translated for the first time by James Legge.

^2^The English version was published by The Commercial Press in 2006, translated for the first time by Jan Julius Lodewijk Duyvendak.

^3^For details, please visit http://www.gov.cn/shuju/2022-02/28/content_5676015.htm.

^4^For details of the regulations, visit https://www.mee.gov.cn/gkml/sthjbgw/qt/201808/t20180807_447822.htm.

^5^Additionally, the data surrounding industrial sector in the statistical yearbook mainly reveal industrial enterprises above designated size in China.
